# COVID-19 and Physical Distancing Measures: Experience of Psychiatric Professionals in Europe

**DOI:** 10.3390/ijerph19042214

**Published:** 2022-02-16

**Authors:** Hélène Kane, Jade Gourret Baumgart, Emmanuel Rusch, Gaëtan Absil, Jocelyn Deloyer, Wissam El-Hage, Donatella Marazziti, Andrea Pozza, Johannes Thome, Oliver Tucha, Wim Verwaest, Laurence Fond-Harmant, Frédéric Denis

**Affiliations:** 1 EA 7505 Éducation, Ethique, Sante, Université François-Rabelais, 37020 Tours, France; jadegourretbaumgart@gmail.com (J.G.B.); emmanuel.rusch@univ-tours.fr (E.R.); frederic.denis@univ-tours.fr (F.D.); 2 Haute École Libre Mosane, Département Social, Laboratoire D’anthropologie Sociale et Culturelle (LASC), Université de Liège, 4000 Liege, Belgium; g.absil@helmo.be; 3 Centre Neuro Psychiatrique St. Martin (CNP St Martin), 5100 Namur, Belgium; jocelyn.deloyer@saintmartin.ofc.be; 4 CIC 1415, U 1253 iBrain, Institut National de la Santé et de la Recherche Médicale (INSERM), Centre Hospitalier Régional Universitaire (CHRU), 37000 Tours, France; wissam.elhage@univ-tours.fr; 5 Department of Clinical and Experimental Medicine, Section of Psychiatry, University of Pisa, 56126 Pisa, Italy; dmarazzi@psico.med.unipi.it; 6 UniCamillus, Saint Camillus International University of Health and Medical Sciences, 00131 Roma, Italy; 7 Department of Medical, Surgical and Neuroscience Sciences, University of Siena, 53100 Siena, Italy; andrea.pozza@unisi.it; 8 Department of Psychiatry and Psychotherapy, University Medical Centre Rostock, 18147 Rostock, Germany; johannes.thome@med.uni-rostock.de (J.T.); oliver.tucha@med.uni-rostock.de (O.T.); 9 Centre Hospitalier Neuro-Psychiatrique, 9012 Ettelbruck, Luxembourg; wim.verwaest@chnp.lu; 10 Agence de Coopération Scientifique Europe-Afrique (ASCAE), Grand Duché de Luxembourg, 2010 Luxembourg, Luxembourg; fond.harmant@gmail.com; 11 LEPS, Université Sorbonne Paris Nord, UR 3412, 93430 Villetaneuse, France

**Keywords:** COVID-19, digital technologies, mental health, professional healthcare practices, psychiatry

## Abstract

A The COVID-19 pandemic has had a considerable impact on the organization of psychiatric care. The present study examines how care professionals experienced this period and faced these new constraints weighing on their professional practices. Based on a qualitative research methodology, 13 group interviews with healthcare professionals working in psychiatric wards were conducted in five countries in western Europe. To complement this, 31 individual interviews were carried out in Belgium and France. Public health measures hindered certain therapeutic activities, jeopardized communication, and obliged healthcare professionals to modify and adapt their practices. Confronted with a transformation of their usual roles, healthcare professionals feared a deterioration in the quality of care. Impossible to continue in-person care practices, they resorted to online videoconferencing which went against their idea of care in which the encounter holds an essential place. The lockdown contradicted efforts to co-build care pathways toward readaptation, social reintegration, and recovery, thus reviving the perception of psychiatric hospitalization based on isolation.

## 1. Introduction

The COVID-19 pandemic forced psychiatric and mental healthcare services to adopt a set of public health measures aimed at limiting the risk of the spread of the virus. Indeed, as other past pandemics have shown us, these wards are particularly at risk of becoming sources of contamination due to group living arrangements and the vulnerability of the patients [1,2]. Adopted measures include wearing face masks, respecting minimal physical distancing, the repeated disinfection of surfaces, regularly airing out spaces, screening tests, stopping all group activities, closing wards, suspending outings and visits from relatives, prohibiting any circulation between wards, and setting up certain activities on a remote basis.

In this context of sanitary crisis, mental healthcare professionals have been confronted with a challenge that is two-fold: preventing the risk of contamination [3,4] and ensuring a continuity of care [5]. The relational dimension of care is central in psychiatry, a medical domain where communication is the main means of care [6,7]. The measures taken to prevent the transmission of COVID-19 have greatly jeopardized the continuation of common therapeutic practices, whether these be consultations, group therapy, or social activities. Physical distancing measures have limited communication, particularly non-verbal communication [8], or the perception of scents [9]. Physical contact has been banned by these measures, therefore curbing gestures of welcome, affection, and reassurance [10]. In addition, healthcare professionals, particularly nurses, have been mobilized to conduct new and repeated activities linked to hygiene, tests, and surveillance against COVID-19, which takes away from the time dedicated to their patients.

When these new occupational constraints were implemented, the working conditions were already strained in psychiatry and mental health contexts in many countries before the onset of the pandemic [11,12]. Indeed, in addition to the dual hardship of mental and physical work inherent to working in psychiatry and mental health [7], difficulties due to a lack of resources prevented healthcare professionals from working in conditions that would ensure quality care. Professionals in the field regularly pointed out these difficulties, adding that recruiting new volunteers to be trained to work in psychiatry and mental health has been complicated for several years [13]. This is all the more worrying, as the mental health care needs of the European population have been increasing in recent years, and researchers and professionals have announced a “psychiatric wave” following the COVID-19 health crisis [14], suggesting that there is a new work overload for working professionals.

In this article, we consider how psychiatric healthcare professionals experienced this period and how they have adapted to the coercive measures adopted in hospital wards. Our focus here will notably highlight their perceptions of care, which is considered to be relational work [15]. Forms of care, defined as “a set of words and acts responding to values and aimed at supporting, helping, and accompanying persons who are fragilized in their body and mind” [16], have indeed been significantly impacted by physical distancing measures. Reorganizing wards in response to the new demands has also re-fashioned the roles of psychiatric healthcare professionals and the way in which they identify themselves in terms of intra, inter, and trans-professional interactions [17]. We propose analyzing how healthcare practices have been affected by public health measures and how the adaptive efforts carried out call into question both the values attributed to care in psychiatry and those associated with the roles of healthcare professionals.

## 2. Materials and Methods

### 2.1. A Qualitative Methodology

This work is based on a qualitative research methodology using in-depth group and individual interviews. These interviews were carried out within the PsyGipo2C research project financed by the French National Research Agency and the Centre Val de Loire Region. The PsyGipo2C project takes a particular interest in the impact of COVID-19 on mental health and psychiatry professionals in Europe. Our research protocol received the approval of the ethics council of each of the partner countries in this study: Germany, Belgium, France, Italy, and Luxembourg.

Stimulating collective reflection, the group interviews aimed to understand how psychiatric healthcare professionals faced the sanitary crisis linked to the COVID-19 pandemic and the experience and observations they drew from it for their therapeutic practices. These group sessions allowed various points of agreement and disagreement to emerge about shared experiences [18]. Organized by the study partners in each country, the groups were composed of four to ten participants from different professions working in the same ward. In addition to producing data, the group discussions served to share experiences and to build interprofessional reflexivity.

Individual interviews were also carried out in two francophone partner countries: Belgium and France. These individual interviews helped to situate the elements that were highlighted in group discussion within singular, individual experiences, and personalized thought processes.

An interview protocol for individual and group interviews were designed after a systematic review of the international literature [19] and following exchanges with members of the scientific board who also work in psychiatric wards [20]. These questionnaires were designed with three main themes: organizational adaptations and modifications, use of follow-up methods integrating digital tools, and personal and professional experiences related to the crisis. Group interviews were organized by our partners in Germany, Belgium, Italy, and Luxembourg. The research officer and senior research officer participated in the group interviews and oversaw the study in order to harmonize the methodological approaches. They were involved in the research project from the beginning of the protocol, they have degrees in social sciences, and they were able to answer all questions related to the research project. They also carried out individual interviews. No fixed question order was assigned in order to allow for a freer dynamic during the discussion, and the questions themselves were adapted to contextual variations and the singular experiences of the participants. Interviews were anchored in the reality of the situations encountered while also encouraging a reflection on these experiences [21] in order to avoid the pitfall of general and preconceived remarks about the sanitary crisis. With an approach stemming from experiential social sciences [22], we have taken care to situate these experience narratives according to their specific context.

These interviews were carried out between March and May 2021, about one year after the beginning of the COVID-19 pandemic and at the onset of many public health measures that changed over time. At the time of the interviews, the vaccination rate was very low in European countries, and most public health measures were maintained in psychiatric wards and within the general population. In order to carry out these interviews, the authors were required to adopt preventive measures: wearing masks, washing their hands regularly, using hydro-alcoholic sanitizer, keeping a distance of two meters between them, and airing the room for 10 min between interviews.

### 2.2. Interviewee Profiles

Participants who made an informed choice to participate were recruited by study partners on a volunteer basis [23]. For this convenience sampling, special attention was given to include a diversity of professions and professional experiences in order to better grasp how the impact of the public health measures could vary according to socio-professional cultures. The professionals who participated in the individual interviews did not know the interviewer and were not expected to meet with him afterwards. In the case of face-to-face interviews, consent was obtained from all participants in writing.

In all, 13 group interviews (GI) and 31 individual interviews (II) were carried out with healthcare professionals working in psychiatric wards, for a total of 96 participants (see Table 1). Due to the sanitary context, group interviews were conducted remotely via videoconference. However, the majority of the individual interviews (24/31) were carried out in person in order to foster optimal communication conditions. Individual interviews lasted between 28 and 71 min, resulting in an average duration of 54 min. Group interviews lasted between 43 and 88 min, resulting in an average duration of 59 min. Among the participants, 64 were women and 32 were men, correlating to the overrepresentation of women in certain professions, notably social work and nursing. At the time of the pandemic, all of the professionals who were interviewed were working in psychiatric and mental health services. Some of them temporarily worked from home or were assigned to wards to care for patients with COVID-19.

### 2.3. Interview Analysis

Individual and group interviews were recorded by dictaphone and were fully transcribed before being imported in the NVivo© analytical software program (QSR International, Doncaster, Australia). Group interviews in German and Italian were transcribed and then translated into French by the concerned partners. The process of retranslating the content from the target language to the source language in literal terms was carried out through a back-translation process [24]. An iterative process was used to draw up a thematic analysis tree in French in collaboration with the partners [25]. In other words, the authors made on-going and progressive adjustments to the thematic analysis depending upon the research question and the interview data [26]. In the first step, the two authors coded two group interviews and five individual interviews in order to verify the coding correlation. Then, the thematic analysis tree was adjusted to provide more detail to certain categories. Then, the analysis tree was validated by all of the partners, and the two researchers proceeded to code the entire corpus of interviews.

## 3. Results

Four main thematic axes highlighting the most significant aspects of the psychiatric experiences of the healthcare professionals, and merit analysis is adapted here. These axes are the cessation of certain therapeutic activities, the adoption of new roles, questions surrounding the relevance of the public health measures, and the ways in which digital technology was used to adapt to the situation.

### 3.1. Ceasing Certain Therapeutic Activities

The onset of the pandemic and the deployment of strict measures, particularly during the lockdown periods, considerably affected the continuity of therapeutic activities in psychiatric wards. In order to limit the risk of contamination, preventive measures were put into place within these wards: a reduction in the number of hospitalized patients; the closure of certain services; the interruption of out-patient consultations; the suspension of external activities, outings, and visits; and the establishment of special units, in which wards were divided into smaller units to avoid circulation between wards. The professionals described their impression of these “frozen” and “idle” wards and described feeling as if they were on stand-by and as if “it were a long drawn-out month of August”; for some, certain wards were like “phantoms”, at a complete standstill.

“*The pandemic blocked the ward where I work. […] for us, agents and professionals, it was very hard to feel useless and to not be able to do our job*.”

(Woman, psychologist, GI 10, Italy)

Certain activities that had been interrupted were important in the therapeutic process. Their suppression forced healthcare professionals to propose alternative activities to patients so as to not leave them with “nothing to do”. However, these supplementary activities were not as therapeutically relevant. Often, it was no longer possible to rely on the daily activities that provided patients with structure for participation. Hospitalized patients were no longer allowed to do dishes, set the table, or do laundry. It was presumed that the patients would not know how to apply the new standards of hygiene that these activities now required. More broadly, group activities–often used in psychiatric therapy–were frequently proscribed, drastically limiting the realm of therapeutic possibilities.

“*It is true that we have patients for whom our goal is to re-socialize, to rehabilitate them through group work notably. All of this was stopped. […] It is true that our range of care treatment was amputated of its group dynamic…whereas, in psychiatric wards, this is an important aspect*.”

(Man, psychologist, GI 5, Belgium)

Another difficulty was the closure of certain social, administrative, and care services that often work in close collaboration with psychiatry. Social work and insertion projects were suspended; professionals could hardly work on organizing outings. They had the impression that their patients were blocked in a sort of “in between” and that they were “sidelined”; they could no longer meet with family members to prepare any returns home. The fact that activities outside of the care structure became impossible contributed to “disconnecting” patients from the outside world even though the professionals continued to search for ways to maintain these connections.

In addition to the suppression of activities, mandatory physical distancing and face masks hindered communication with patients. This led to a partial loss of non-verbal communication and some expressivity, making it more difficult to establish the trust that is necessary in psychiatric care.

“*The contact with the patients changes in the first instance, when you put up a plastic screen between patients, between patients and visitors, like we do, there is always a certain separation that is palpable in the discussion*.”

(Man, psychiatrist, GI 1, Germany)

Wearing face masks changed methods of communication, particularly in terms of expressing emotions and affect. Methods of communication had to be adapted since body language is at the heart of the therapeutic act in psychiatry.

“*I would say that wearing a mask, in everyday care, is very complicated because patients can’t see our emotions. What we can convey, we are almost obliged to put everything in quotes and “overact” […] overact with our eyes to express emotions we could have conveyed through a smile. […] And we work a lot with our bodies, with our presence*.”

(Man, psychologist, GI 7, France)

The public health measures led to an erasure of the body in the care relationship. Banning physical contact notably impeded gestures of comfort or expressions of empathy. Care was thus deprived of its bodily and tactile dimension, which is valued by nursing cultures of “care”.

“*There are patients who react well to touching, now that is all over. The fact of holding back, from holding a hand, from letting someone rest their head on your shoulder because they want to lean over, it’s all over. For elderly people, who don’t see their families anymore, it was quite damaging. […] It was complicated to tell them: “No, we can’t touch you*.”

(Woman, nurse, GI 8, France)

Physical distancing led to a modification in how interactions were performed in order to preserve the empathy and sincerity necessary for care. Restructuring spaces in order to ensure physical distancing hindered formal exchanges, but also less daily interactions. Distancing also concerned patients among themselves, affecting their communication and the relationships they could have built.

### 3.2. Taking on New Roles

Organizational changes affected caregivers and their roles, leaving them less available for mental health and psychiatric care. Some professionals were moved to units that were in charge of COVID-19 patients.

“*We stayed closed down for three months to make room for COVID-19 wards. For me, I was catapulted into the electroconvulsive therapy unit, where I had never worked; luckily the anesthesiologists helped me out, it was really tough*.”

(Woman, nurse, GI 9, Italy)

The activity of caregivers who remained in their units was also reshaped in response to COVID-19. Nurses and nursing assistants were occupied with hygienic tasks, Polymerase Chain Reaction tests (PCR), and applying and monitoring preventive measures. These healthcare professionals were the most impacted by the excess work linked to the daily struggle against the risk of contamination. In some cases, occupational therapists, special-needs workers, and social workers came to help them and were thus forced to neglect their own usual work. This shift in work tasks led professionals to take on new roles that far from their habitual skills. Due to this, many felt a loss of meaning and sometimes difficulty in finding their place.

“*Sometimes, we don’t know how to make ourselves useful, so there are moments when we feel useless, not knowing what to do, where to go… […] An uncomfortable position to be in. […] Sometimes, it was like being a student-intern*.”

(Woman, psychomotor therapist, GI1 3, Luxembourg)

Being required to monitor preventive measures absorbed the energy of many professionals. Many had the feeling that they were playing a controlling role, that of a “policeman” or a “guard”, which they did not feel was relevant to their prerogatives.

“*It is true that I took respecting these measures to heart, because I felt as if I was also in danger. During this first wave, I don’t remember having done any psychiatry, I felt more in a disciplinary role… “Be careful, don’t touch everything, wash your hands, stay at a proper distance” … I think I must have repeated that four hundred times*.”

(Woman, nursing assistant, GI 4, Belgium)

The need to constantly monitor preventive measures, to incessantly repeat things to patients who had difficulties integrating them due to their mental troubles, interfered with care and treatment. Some professionals did, however, appreciate trying other functions.

“*I was part of a team of caregivers for a month, and I found the experience quite enriching, very positive. […] I could see a bit of what my colleagues do, and I think that allowed us to create a stronger professional bond, so…seeing a bit of what they deal with every day*.”

(Woman, social worker, GI1 2, Luxembourg)

If most professionals felt that they “did a good job” in applying the hygiene rules, some feared that they would lose the “human” contact of care. The communication with their colleagues was also hindered by the public health measures, and some noticed less coordination during care. Others felt as if they no longer worked in mental health or psychiatry, but rather that they were only scratching the surface and were only doing busy work. Confronted with the sanitary crisis, they lowered their therapeutic ambitions, preferring to postpone costly psychological activities.

“*We had to relax, contain our anxiety […] Psychological care, well that was pushed to the back burner, it required a lot of energy that we didn’t have at that time. […] For me too, sometimes, it was nice to sing a karaoke or to go on a genuine walk outside, rather than guide my self-affirmation group, which requires a lot, which gets patients to work on their problems, because there was a lot of fatigue…*”

(Woman, psychologist, GI 7, France)

Affected by new professional constraints or sometimes to difficulties to relax and reenergize in their personal life, healthcare professionals shared the “fatigue” felt by their patients concerning the situation that pushed them to decrease the intensity of their therapeutic work.

In confined circumstances where many outside activities were forbidden, special relationships–less focused on therapy–were paradoxically built. Being together over a long period of time and sharing the experience of this unprecedented crisis led to a confusion regarding familiar and friendly relationships. This impression was strengthened by the fact that the professionals themselves were affected by the restrictions that impacted their own social lives. Indeed, many felt that they better understood what their patients were going through. As medical appointments were only rare, authorized encounters, patients and professionals tended to show a greater tendency to treat them as ordinary social encounters.

### 3.3. Questioning the Relevance of Public Health Measures

While fearing the risk of contamination outbreaks in their units, certain professionals questioned the relevance of the implemented public health measures due to the impact on care and the mental health of their patients. Many shared the feeling that these measures changed the quality of care and sadly remarked that their patients’ psychological troubles had deteriorated, whether they be hospitalized patients suffering from the restrictions placed on activities or out-patients confronted with the public health measures applied to the general population.

“*We all had our psychotics in out-patient care and who were more or less stabilized, but no longer had any bearings! […] And then we saw them come back to be forcibly hospitalized, because what was keeping them going, providing them with some balance with the outside, was no longer there. […] So, we had, between March and the beginning of June, 30% more of forced hospitalizations than other years […] They let themselves go; psychologically, they are not doing well…*”

(Man, psychiatrist, II, Belgium)

Some professionals admired the resilience of their patients who were facing the imposed restrictions. Nevertheless, many felt that the measures were too strict and unfavorable for mental health. In one Belgian hospital where patients could not go outside, their isolation was felt to be harmful, sometimes described as a “prison” or a “hostage situation”. The professionals were outraged that their patients could not meet their loved ones: for one year, they could only have short visits in an outside lobby, behind Plexiglas. Some professionals felt that the measures, which were stricter for the patients hospitalized in psychiatry units than they were for the general population, were discriminatory toward patients suffering from mental illness.

“*There was no way to re-energize, in fact, for the patients. […] We would ask them to get up only to twiddle their thumbs in a room… Nothing made sense for them. We could really sense a depression settling in the patients. […] I tried to contact a person who was in charge of patients’ rights, to ask him: “How far should we go with this restriction?” [she laughs] because we are killing them…*”

(Woman, specialist educator, II, Belgium)

Several professionals mentioned the excessive application of preventive measures that put the mental health of their patients in danger. Facing this risk, certain professionals tried to negotiate the margins of action and freedom for their patients; others transgressed the rules when the “psychological survival” of their patient seemed to be threatened. Some professionals, who felt that they were personally at risk of developing a severe form of COVID-19, believed that the public health measures should have been more strictly enforced, but many others disagreed, considering that the impact on mental health would be more damaging compared to the benefits in terms of limiting the epidemic. Some rules were less respected, as their relevance in terms of risk of infection was not obvious for the healthcare professionals.

“*Elderly people don’t understand you very well already. And now in our unit, we have to wear our mask all day long, I find that difficult. When we have to do consultation visits, we have to get close for them to understand us. We have to do everything in any case, we have a very close contact with the patients*.”

(Man, nurse, GI 3, Germany)

The healthcare professionals sometimes found themselves in situations where they had to explain and enforce measures that they themselves did not adhere to; measures that they found incoherent or even contradictory. Although they had different missions, the different categories of professionals who were interviewed shared the impression that the imposed conditions did not allow them to ensure quality care. Specialist educators regretted that they could not carry out planned activities. Social workers felt that the work they had begun was blocked. Nurses said they no longer had any time to talk with their patients. Psychiatrists could no longer explore certain therapeutic dimensions. The feeling that hospital and working conditions were deteriorating and causing changes in their patients’ health led to a fatigue associated with forms of suffering for the professionals, combining worry, outrage, and discouragement. Some mentioned a feeling of saturation and less mental availability for their patients. These professionals also stressed how much the restrictions of movement imposed upon their patients went against their autonomy. Isolated from the outside world, these patients became more and more dependent upon the care structure. Certain professionals also had the feeling that the rationale behind the sanitary measures to contain the pandemic had overridden psychiatric care and that care ethics were undermined.

“*We focused on the containment of infection. […] We didn’t want to get sick ourselves. […] That is why we did everything we could and accepted certain things that didn’t work. That led to a loss in the quality of care*.”

(Woman, nurse, GI 3, Germany)

The changing public health measures were also harmful for people suffering from mental illness. It was particularly difficult for the professionals to adapt and build some sense of coherency when faced with changing rules.

“*It is true that telling them twenty times a day that they have to put their mask back on really pushes them further back in the corner. […] For things that we would have tolerated in order to create trust and alliance, now we are forced to frustrate them more seriously. This isn’t how we would have done things in psychiatry: we would have figured out another way to do things, taking more time to get the same results*.”

(Woman, nurse, II, France)

The logic behind public health measures–their strict application to avoid contamination–contradicts care that normally comprises flexibility, adaptability, negotiation, and adjustment.

### 3.4. Adapting with Digital Tools and Technology

These constraints pushed the professionals to innovate, notably engaging in activities without “contact” or the transmission of objects. Some used digital tools to compensate for certain activities that were no longer possible. The creativity of some professionals in this domain was thus revealed through the crisis. Some made videos, some organized forums or games on social media, and others held videoconferencing encounters. Many expressed the fact that the crisis provided them the opportunity to “reinvent themselves” despite the restrictions and sometimes rough periods.

“*During the second lockdown we were able to take charge, because we had the experience from the first lockdown, and we did not want to relive the same things. […] We put things into place digitally, we reinvented a new way of working to compensate for this state of depression and to not suffer from the second wave. […] It was a breath of fresh air to work with this group via videoconference, because we were finally in contact and felt useful*.”

(Woman, specialist educator, II, Belgium)

New activities could be proposed thanks to digital tools. However, it was mainly younger professionals who innovated in this way. For the majority, using digital tools was limited to remote consultations or online meetings, which helped to maintain a certain continuity of care despite relational changes. The experiences related to remote consultations, which were unprecedented for many, seem contrasted. Some professionals were at first reluctant toward the idea of remote consultations but were then surprised to discover that they could maintain therapeutic interactions despite the physical distance and a loss of non-verbal communication.

Psychiatrists and psychologists explained that this new experience led them to consider the interest of remote consultations in certain situations. Professionals whose missions demand more close contact with patients, notably nurses, were less favorable toward digital tools. They were especially worried about a certain “dehumanization” that these digital tools would cause, even though the crisis had reduced opportunities for encounters. In addition, considering psychiatric troubles as a rupture in social ties, some participants argued that digital tools would only but weaken social links.

“*Addictions are first and foremost a disease linked to lack of social bonds. So, [sigh], eliminating social bonds, I really don’t see the point or interest. […] Today, there is more and more individuality in our societies and families are split and fragmented. […] We see more and more psychiatric illnesses, so eliminating this bond even more isn’t suitable*.”

(Woman, professional peer support worker, II, France)

Not all professionals considered physical distance as synonymous with social distance. However, all of them were more or less concerned about how digitalizing encounters would impact the therapeutic relationship. The physical encounter established a formal context requiring the presentation of oneself [27] and thus anchored the physical body in space and time. This would diminish with videoconferencing. One psychiatrist related that her patients would be dressed in their pajamas on their beds during the remote consultations, which she described as “regressive”. Other professionals mentioned patients more frequently missing appointments due to their lack of spatial-temporal understanding and the fact that they no longer had a material appointment “card”. Without the “physical” commitment of the face-to-face encounter, the involvement in the care process did not benefit from the same structure [28], both for the patients and for the professionals. More broadly, many professionals had the feeling that videoconferencing did not allow them to work with the same intensity.

“*We know well that in therapy, there is all the infra-verbal… all of these gestures of empathy that go through the body, through the eyes, a smell, and that can evoke very archaic things. […] When I say archaic, it is in terms of becoming a baby of sorts, the baby is a sensorial being… […] There is something like that, something intersubjective that is communicated through the eyes, and that has to be embodied*.”

(Woman, psychiatrist, II, France)

Other professionals remarked that their “remote” consultations were shorter, sometimes limited to simply checking in or renewing a prescription. Their reluctance to tackle certain sensitive topics or to work on traumatic experiences was also founded on the fear of causing discomfort for their patient whom they would not be able to help due to the fact that they were not present.

## 4. Discussion

Based on in-depth interviews, our study documents the experiences of professionals working in psychiatry and mental health who had to adapt to the COVID-19 pandemic. At the time of the interviews, many public health measures were still being applied in the concerned countries. These measures caused most of our interlocuters fatigue and discouragement. Tainted by the morosity of the period, their responses are to be considered in light of this singular context, which is marked by the imposition of curfews, closures, travel restrictions, and constraining measures in their own units. This work provides reflexive feedback on a year of work in psychiatry during COVID-19, with its narrative necessarily situated in a singular context [29].

### 4.1. Different Experiences from One Context of Professional Practice to Another

In all of the countries in this study, the professionals were confronted with an increase in their workload, which was linked to reorganization and additional hygiene-related tasks as well as to stress generated from uncertainty. Our study is consistent on this point with data from the scientific literature [19]. Depending on the human resources at their disposal, wards were able to reorganize with more or less difficulty in order to absorb the excess work and to compensate for COVID-19-related work leave. Structures with better human resources, including diversified teams with specialist educatosr, occupational therapists, or psychomotor specialists, were able to distribute certain tasks to these professionals who were limited in their usual activities. These professionals, although frustrated because they could no longer do their normal work, drew satisfaction from the fact that they were included in a collective response to the crisis situation.

Applying public health measures proved to be somewhat strict depending on the units, leaving healthcare professionals little room to maneuver in continuing their activities. Certain professionals who considered the imposed restrictions untenable saw their distress increase because it was impossible for them to adjust to the public health measures according to different situations and/or the mental health profiles of their patients. Healthcare professionals working within establishments for the dependent elderly also suffered from this distress due to imposed restrictions [30].

Although the professionals encountered understood the importance of public health measures to protect their patients from COVID-19, their attention and sensitivity toward mental health led them to think about these measures and sometimes manifest their disagreement or even commit acts of disobedience. It is not our aim here to question the relevance of the public health measures or the way in which these professionals carried them out, but rather to reveal how much these measures caused the professionals interviewed here to face contradictory demands and ethical dilemmas, something that has also identified in other research work [31]. In addition, many felt alone against the measures decided by their hierarchy, which in some cases, were impossible to apply due to infrastructural constraints. In the face of this common challenge, some professionals were more affected than others, depending on their missions and the available resources, but also depending on their own sensitivity. Nurses and nursing assistants, who had to deal with a significant increase in their workload, seem to have struggled with the tension imposed by having to respect social distancing while ensuring the continuity of daily care. The experience of these healthcare professionals during the pandemic has been infrequently documented. Yet, they were particularly hampered in maintaining fragmented patient care relationships, which are at the heart of their profession [15]. They were also more exposed to patient difficulties on a daily basis. The informal aspect of their work, which contributes to the patient feeling more human over the course of daily interactions [29], was particularly impacted by public health measures. These measures did not always allow the therapeutic dimensions of daily activities such as meals [32]. Comparatively, psychology or psychiatry consultations would be easier to conduct with social distancing or remote consultation. As documented in the literature, these healthcare professionals turned more and more to remote consultations during the pandemic [33].

### 4.2. Distress over Measures Going against Professional Values

Beyond these major contextual differences, the public health measures jeopardized the continuity of care for everyone in psychiatry. The professionals experienced interruptions in care and communication weakened by social distancing and masks [8]. As a result, the professionals’ own conceptions of healthcare, at the heart of which the encounter and communication take a central role, were put to the test. Beyond varying points of view, the COVID-19 crisis brought common values of care and accompaniment to the surface [16].

Unable to pursue their usual role, certain professionals were frustrated in their perceptions of the meaning of their work. Their activities were restricted, whereas they are used to a certain independence and autonomy in their work, as the tasks at hand are not standardized and thus require initiative and personalized care [7]. This situation where they were unable to practice their profession according to these rules and expectations resulted in “occupational identity suffering” [34]. Aggravated by the crisis, the burden of the perceived impossibility of accomplishing quality relational work in psychiatry had already been observed in contexts with insufficient human resources [7,35]. Some professionals acknowledged that this situation had affected their own mental health and that as a result, they were less mentally available to ensure care for their patients. Our study shows that psychiatric healthcare professionals, although not on the frontline of COVID-19 care, have been put to the test by the pandemic in particular. This is consistent with research showing that healthcare professionals are exposed to, and at risk for, mental health problems [36,37]. The way in which the professionals accepted temporarily taking on new roles was indicative of the relationship that they had with their hierarchy, torn between a certain respect for decisions made and a feeling that the challenges faced during their duties were not acknowledged. In some cases, the distress of not being able to uphold quality care was aggravated by the feeling that these difficulties were not recognized by their hierarchy. Scientific literature data underlines the importance of ensuring a continuity of care in times of crisis in order to avoid a deterioration in the mental health of patients hospitalized with psychiatric-related problems [38].

Restrictions on visits and outings undercut the professionals’ efforts to foster recovery. Indeed, the recovery paradigm supposes that psychiatric service users be considered as the driving force in their own lives, as fully fledged citizens. Psychiatric wards should be designed in such a way as to support patient autonomy rather than perpetuate the conventional role of the patient [39]. Isolation and lockdown represent this backward movement, undermining any steps taken toward social inclusion and empowerment. Restrictions on leaving the wards have revived ideas of confinement in asylums [40], whereas today professionals tend to favor opening units, de-institutionalization, and out-of-hospital care.

### 4.3. The Body in the Care Relationship and Remote Care

Experiencing social distancing measures led the professionals to reconsider the importance granted to the body in the care relationship. This “place” given to the body is defined differently according to professions. Occupational cultures thus contribute to building different forms of embodiment [41]. While all consider that nothing can replace the in-person encounter–considered to be “true” encounters–paramedics and social workers have shown more worry about the development of digital tools in psychiatric care, fearing a “de-humanization” of care. The way in which these different professionals apprehend remote care echoes the distribution of work tasks that necessitate a relative physical proximity with patients [42].

Nevertheless, this unprecedented context has spurred a reassessment of “relational psychological care” in the medical world, which has remained hostile to digital technology, in order to prevent patients from relapsing [43]. The use of digital technology and notably videoconferencing was paradoxically embraced as a potential form of exchange when physical encounters were forbidden as well as an impoverished form of communication. Psychologists and psychiatrists were more easily convinced by the possibilities of videoconferencing. Experimenting with the limits of remote consultations and enduring a few setbacks, they came to understand the need to reestablish the right relational distance by providing a new framework of care [44]. New uses of digital technology have triggered a recalibration of therapeutic relationships between asymmetry and reciprocity [45]. The experience of videoconferencing allowed many professionals to overcome some of their initial hesitations [46].

However, contrary to what is mentioned in certain publications, the healthcare professionals interviewed for this study did not consider that this virtual space of remote consultation could lead to building new forms of intimacy in the therapeutic relationship [47]. The COVID-19 crisis has nevertheless paved the way for the development of hybrid care practices, integrating the advantages of remote consultation as a complement to face-to-face encounters [48].

### 4.4. Limitations

This work does not pretend to compare countries, which would suppose a systematic comparison of the specificities in terms of health policies, psychiatric healthcare organization, and the rights of hospitalized patients. It does, however, analyze, based on multi-situated investigations, the experience of professionals in singular contexts, revealing the importance of professional organizations and the means that they had at their disposal to confront the sanitary crisis.

We can add the limitations that are inherent to all qualitative studies, which are related to small sample size, potential response bias, and self-selection bias. Furthermore, despite our efforts to construct an exhaustive interview guide, it is not excluded that some grey areas were not uncovered during our investigation.

## 5. Conclusions

The COVID-19 pandemic forced the reorganization of psychiatric and mental health services and led health professionals to adapt to new situations and contexts that hindered the continuity of their therapeutic work. Confronted with the dual challenge of managing the epidemic and ensuring the continuity of care and support for patients, healthcare professionals adopted postures and attitudes of renunciation, resistance, and adaptation. Physical distancing measures obliged them to experience relational and organizational situations, in which they re-evaluated the importance of physical co-presence in care, but also the opening of their units to the outside world. The tension, and often the contradiction, that exists between therapeutic requirements and these imperatives of health security and social control, accounts for the conflictual mode and misunderstandings of daily life within hospital work organizations [49]. This experience made them question their roles and missions as caregivers, brushing up against the limits of their values and ethics. As a result of their shared experience of pandemic constraints, they became particularly sensitive to what their own patients were going through. Confronted with situations reminiscent of asylum confinement [49], they implemented innovate and resilient strategies in order to preserve the dignity and mental health of their patients.

## Figures and Tables

**Table 1:** Distribution of group and individual interview participants according to profession in psychiatric wards.

Profession	Germany			Belgium			France				Italy			Luxembourg		
	GI 1	GI 2	GI 3	GI 4	GI 5	II	GI 6	GI 7	GI 8	II	GI 9	GI 10	GI 11	GI 12	GI 13	
Psychiatrists	2	-	-	-	1	1	-	-	-	4	1	2	-	2	-	13
Psychologists	2	-	-	-	1	2	-	2	-	2	1	2	-	-	-	12
Psychiatric nurses	-	4	5	2	4	2		1	2	4	3	-	-	1	2	30
Nursing assistants	-	-	-	1	1	-	2	-	1	1	-	-	-	-	-	6
Social workers (specialist educator)	-	1	-	-	1	2	-	1	1	2	1	-	-	1	-	10
Occupational therapists, physical therapists	-	-	-	1	1	2	-	-	-	-	-	-	-	-	3	7
Other (secretaries, assistants, students)	1	-	-	-	1	4	2	-	-	5	-	-	5	-	-	18
Total	5	5	5	4	10	13	4	4	4	18	6	4	5	4	5	96

## Data Availability

Data are fully available and will be shared upon request to H.K.

## References

[1] Kelly B.D. (2020). Plagues, pandemics and epidemics in Irish history prior to COVID-19 (coronavirus): What can we learn?. Ir. J. Psychol. Med..

[2] Esterwood E., Saeed S.A. (2020). Past Epidemics, Natural Disasters, COVID19, and Mental Health: Learning from History as we Deal with the Present and Prepare for the Future. Psychiatr. Q.

[3] Kreuzer P.M., Baghai T.C., Rupprecht R., Wittmann M., Steffling D., Ziereis M., Zowe M., Hausner H., Langguth B. (2020). SARS-CoV-2 Risk Management in Clinical Psychiatry: A Few Considerations on How to Deal With an Unrivaled Threat. Front. Psychiatry.

[4] Li S., Zhang Y. (2020). Mental healthcare for psychiatric inpatients during the COVID-19 epidemic. Gen. Psychiatr..

[5] Sangalli L., Fernandez-Vial D., Moreno-Hay I., Boggero I. (2021). Telehealth Increases Access to Brief Behavioral Interventions in Orofacial Pain Clinic during COVID-19 Pandemic: A Retrospective Study. Pain Med..

[6] Thomas J. (2010). Les lieux de communication dans l’urgence psychiatrique. Communication et Santé: Enjeux Contmporains.

[7] Estryn-Behar D., Duville N., Menini M.L., Foll S., Nézet O., Bocher R. (2006). Mots à maux… Expression de la souffrance chez les soignants en psychiatrie. Étude comparative en France et dans trois autres pays européens. Ann. Med.-Psychol..

[8] Robin-Poupard F. (2020). Distanciation sociale, masques et visioconférence: Être thérapeute au temps de la COVID-19. J. Psychol..

[9] Hanon C. (2019). Le nez du psychiatre ou comment l’odeur est nécessaire à la relation thérapeutique en psychiatrie?. Fr. J. Psychiatry.

[10] Paul E., Crommelinck B., Decker M., Doeraene S., Kaisin P., Lallemand B., Noël C., Van Ypersele D. (2020). Impacts de la crise du COVID-19 sur un hôpital psychiatrique pour enfants et adolescents. Cah. Crit. Thérapie Fam. Prat. Réseaux.

[11] Petho B. (2008). Recent crisis of psychiatry in the context of modern and postmodern science. Psychiatr. Hung..

[12] Bourdeux C. (2003). Psychiatry is in crisis!. Soins Psychiatr..

[13] Lunn B. (2011). Recruitment into Psychiatry: An International Challenge. Aust. N. Z. J. Psychiatry.

[14] Han R.H., Schmidt M.N., Waits W.M., Bell A.K.C., Miller T.L. (2020). Planning for Mental Health Needs During COVID-19. Curr. Psychiatry Rep..

[15] Bautzer É.R. (2016). Chapitre 2. Une approche sociologique du soin comme travail relationnel. J. Int. Bioethique D’ethique Sci..

[16] Saillant F., Gagnon E. (1999). Vers une anthropologie des soins?. Anthropol. Sociétés.

[17] Abbott A. (1993). The sociology of work and occupations. Annu. Rev. Sociol..

[18] Baribeau C., Germain M. (2010). L’entretien de groupe: Considérations théoriques et méthodologiques. Rech. Qual..

[19] Gourret Baumgart J., Kane H., El-Hage W., Deloyer J., Maes C., Lebas M.C., Marazziti D., Thome J., Fond-Harmant L., Denis F. (2021). The Early Impacts of the COVID-19 Pandemic on Mental Health Facilities and Psychiatric Professionals. Int. J. Environ. Res. Public Health.

[20] Thome J., Deloyer J., Coogan A.N., Bailey-Rodriguez D., da Cruz E.S.O.A.B., Faltraco F., Grima C., Gudjonsson S.O., Hanon C., Hollý M. (2020). The impact of the early phase of the COVID-19 pandemic on mental-health services in Europe. World J. Biol. Psychiatry.

[21] Vermersch P. (2010). L’entretien D’explicitation.

[22] Heinich N. (2006). Vers une science sociale de l’expérience. Rev. MAUSS.

[23] Fond-Harmant L., Kane H., Gourret Baumgart J., Rusch E., Breton H., El-Hage W., Deloyer J., Lebas M.C., Marazziti D., Thome J. (2021). International professional practices in mental health, organization of psychiatric care, and COVID-19: A survey protocol. PLoS ONE.

[24] Ozolins U. (2009). Back translation as a means of giving translators a voice. Transl. Interpret..

[25] Bardin L. (2013). L’analyse de Contenu.

[26] Olivier de Sardan J.-P. (2001). Le “je” méthodologique, implications et explicitations dans l’enquête de terrain. Rev. Française Sociol..

[27] Goffman E. (1973). La Présentation de Soi. La Mise en Scène de la Vie Quotienne I.

[28] Goffman E. (1974). Frame Analysis: An Essay on the Organization of Experience.

[29] De Villers B. (2016). Vers une signification anthropologique des «dimensions informelles» des soins en psychiatrie. Cercle Herméneutique.

[30] Langlois E., Abraham M. (2021). L’appropriation de la télémédecine dans les EHPAD: Entre contraintes organisationnelles et engagements individuels. Rev. Française Aff. Soc..

[31] Russ M.J., Sisti D., Wilner P.J. (2020). When patients refuse COVID-19 testing, quarantine, and social distancing in inpatient psychiatry: Clinical and ethical challenges. J. Med. Ethics.

[32] Amadei F. (2021). Le repas thérapeutique en hôpital de jour de pédopsychiatrie à l’épreuve des contraintes sanitaires: Thérapie institutionnelle et COVID-19. Ann. Médico-Psychol. Rev. Psychiatr..

[33] Kane H., Gourret Baumgart J., El Hage W., Deloyer J., Maes C., Lebas M.C., Marazziti D., Thome J., Fond-Harmant L., Denis F. (2021). Uses of digital technologies in the time of COVID-19: Opportunities and challenges for professionals in psychiatry and mental health care. JMIR Hum. Factors.

[34] Viviers S. (2016). Souffrance et stratégies défensives dans le travail de conseillers d’orientation en milieu scolaire: L’identité professionnelle en question. Éducation Vie Trav..

[35] Cintas C. (2009). Pénibilité du Travail en Hôpital Psychiatrique. *Perspect. Interdiscip. Trav. St. 11-1*. https://journals.openedition.org/pistes/2283?lang=en.

[36] Raudenská J., Steinerová V., Javůrková A., Urits I., Kaye A.D., Viswanath O., Varrassi G. (2020). Occupational burnout syndrome and post-traumatic stress among healthcare professionals during the novel coronavirus disease 2019 (COVID-19) pandemic. Best Pract. Res. Clin. Anaesthesiol..

[37] El-Hage W., Hingray C., Lemogne C., Yrondi A., Brunault P., Bienvenu T., Etain B., Paquet C., Gohier B., Bennabi D. (2020). Health professionals facing the coronavirus disease 2019 (COVID-19) pandemic: What are the mental health risks?. Encephale.

[38] Dumais Michaud A.-A., Lemieux A.J., Dufour M., Plante L., Crocker A.G. (2022). COVID-19 et pratiques professionnelles dans les milieux institutionnels fermés. Sante Publique.

[39] Jouet E., Greacen T. (2012). Pour des Usagers de la Psychiatrie Acteurs de Leur Propre Vie. Rétablissement, Inclusion Sociale, Empowerment.

[40] Foucault M. (1976). Histoire de la Folie à L’âge Classique.

[41] Csordas T.J. (1990). Embodiment as a Paradigm for Anthropology. Ethos.

[42] Peneff J. (1992). L’Hôpital en Urgence. Etude par Observation Participante.

[43] Bocher R., Jansen C., Gayet P., Gorwood P., Laprévote V. (2020). Réactivité et pérénnité des soins psychiatrique en France à l’épreuve du COVID-19. Encephale.

[44] Ingram D.H., Best K. (2020). The Psychodynamic Psychiatrist and Psychiatric Care in the Era of COVID-19. Psychodyn. Psychiatry.

[45] Girard V., Driffin K., Musso S., Naudin J., Rowe M., Davidson L., Lovell A.M. (2006). La relation thérapeutique sans le savoir. Approche anthropologique de la rencontre entre travailleurs pairs et personnes sans chez-soi ayant une co-occurrence psychiatrique. L’Évolution Psychiatr..

[46] Cowan A., Johnson R., Close H. (2020). Telepsychiatry in Psychotherapy Practice. Innov. Clin. Neurosci..

[47] Kocsis B.J., Yellowlees P. (2018). Telepsychotherapy and the Therapeutic Relationship: Principles, Advantages, and Case Examples. Telemed. J. e-Health.

[48] Mishkind M.C., Shore J.H., Schneck C.D. (2021). Telemental Health Response to the COVID-19 Pandemic: Virtualization of Outpatient Care Now as a Pathway to the Future. Telemed. J. e-Health.

[49] Goffman E. (1968). Asylums: Essays on the Social Situation of Mental Patients and Other Inmates.

